# Therapeutic Physical Exercise for Dysmenorrhea: A Scoping Review

**DOI:** 10.3390/jfmk10010010

**Published:** 2024-12-27

**Authors:** Philippine Rigal, Salomé Bonnet, Ágata Vieira, Alice Carvalhais, Sofia Lopes

**Affiliations:** 1 Departamento de Tecnologias de Diagnóstico e Terapêutica, Escola Superior de Tecnologias da Saúde do Tâmega e Sousa, Instituto Politécnico de Saúde do Norte (IPSN), CESPU, 4585-116 Gandra, Portugal; a28517@alunos.cespu.pt (P.R.); a28541@alunos.cespu.pt (S.B.); agata.vieira@ipsn.cespu.pt (Á.V.); alice.carvalhais@ipsn.cespu.pt (A.C.); 2 Departamento de Fisioterapia, Escola Superior Saúde Santa Maria, Trav. Antero de Quental 173/175, 4049-024 Porto, Portugal; 3 H^2^M—Unidade de Investigação em Saúde e Movimento Humano, Instituto Politécnico de Saúde do Norte, CESPU, CRL, 4760-409 Vila Nova de Famalicão, Portugal; 4 Centro de Investigação em Reabilitação (CIR), Escola Superior de Saúde, Instituto Politécnico do Porto, Rua Dr. António Bernardino de Almeida, 400, 4200-072 Porto, Portugal; 5 Instituto de Ciência e Inovação em Engenharia Mecânica e Engenharia Industrial (INEGI), 4200-465 Porto, Portugal; 6 ESS, Escola Superior de Saúde, Instituto Politécnico do Porto, Rua Dr. António Bernardino de Almeida, 400, 4200-072 Porto, Portugal

**Keywords:** menstrual pain, quality of life, exercise program

## Abstract

**Background:** Dysmenorrhea affects many women of reproductive age. Physical exercise has been used as an effective intervention for pain reduction and to improve well-being. Physiotherapy, involving movement and exercise, can be effective in relieving menstrual pain and provide additional benefits. The aim is to identify therapeutic physical exercise program and exercise protocols used to reduce pain among these women. **Methods:** A scoping review was conducted in accordance with Joanna Briggs Institute’s methodology, using the PCC acronym. Articles were sourced from: PubMed, Cochrane Library, PEDro and ScienceDirect, covering studies published between 1 January 2013 and 30 April 2023, representing the period we considered most appropriate at the time the study was initiated. Qualitative studies, books, book chapters, systematic reviews, meta-analyses and review articles were excluded. Studies were analyzed according to the PRISMA-ScR framework. **Results:** 3325 studies were identified, but only 9 were included. Considerable variation was observed in the types and parameters of the exercise program across studies, including differences in duration, intensity, number of repetitions and series. **Conclusions:** The findings of this study highlight that aerobic training, particularly among women in their 20s, emerged as the most frequently utilized form of therapeutic physical exercise for alleviating menstrual pain in the studies reviewed. This suggests that aerobic exercise may hold significant promise as a non-pharmacological intervention for managing dysmenorrhea.

## 1. Introduction

Dysmenorrhea is defined as “the presence of painful uterine contractions occurring during menstruation” [1]. There are two types of dysmenorrhea: primary and secondary. Primary dysmenorrhea is defined as pain that occurs without underlying organic pathology, typically emerging within three years of menarche [2]. The main symptoms of this type of dysmenorrhea include pain, with or without spasms, mainly below the umbilicus (uterus) and in the lower back, with greater intensity in the first few days of menstruation [3]. Current management strategies for primary dysmenorrhea predominantly rely on pharmacological interventions such as non-steroidal anti-inflammatory drugs or contraceptive pills [4]. Secondary dysmenorrhea, on the other hand, is associated with underlying gynecological conditions, predominantly endometriosis, and this type of dysmenorrhea usually involves premenstrual pain, which can last until the blood flow stops. Treatment approaches vary based on the underlying pathology and surgery may be necessary in some cases [5].

In both cases, the main symptom is pain, mainly uterine, which can extend to the lumbar region. The condition significantly impacts quality of life of affected women, limiting daily activities [6]. In addition, pain can influence woman’s psychological and mental status, perception of pain and sleep quality [7]. The prevalence of dysmenorrhea ranges from 50 to 90% among menstruating women [8,9].

Women affected by menstrual pain are looking for new or complementary management strategies to reduce or eliminate pain, as a substitute for analgesics and/or hormonal contraceptives. Given the limitations and potential side effects of conventional pharmacologic approaches, people are turning to alternatives that are easier to access, more natural, less expensive and potentially with a longer action duration [10]. These include, among others, acupuncture, transcutaneous electrical nerve stimulation, topical application of heat on painful areas of the body (stomach, lower back), dietary modifications, vitamin supplements (particularly vitamin E, which reduces the production of prostaglandin, a hormone associated with menstrual colic), or physical exercise of varying intensities [11,12,13].

When exercising, the body produces endorphins which modulate pain perception through opioid receptor activation [14,15,16].

Exercise has long been recognized as a cornerstone in physiotherapy, offering multifaceted benefits beyond mere physical improvement. When it comes to analgesia and pain control, physiotherapy and exercise can play a crucial role in complementary approaches for reducing symptoms and improving quality of life. In this way, physiotherapists will use different techniques, including therapeutic exercise. In fact, therapeutic exercise aims to restabilize optimal function, activity and participation to maintain a state of physical and mental well-being [17]. The different types of exercises carried out in physiotherapy aim to improve quality of life and increase functionality by improving movement, muscle strengthening, coordination, balance, and general conditioning. These interventions not only improve movement patterns and muscle function but also foster overall resilience, empowering individuals to manage pain more effectively and lead fuller, more active lives [18].

More than a decade ago, physiotherapy for pelvic pain and women’s health was almost non-existent, and a limited number of studies were carried out in this area [19]. Nowadays, it is a more discussed topic, and that is why the role of physiotherapy in women’s health, and specifically in menstrual pain, is increasingly highlighted, with more studies reporting on this subject. This limited interest reflected a broader lack of focus on women’s health issues within both clinical practice and research, leaving a significant gap in evidence-based interventions. However, in recent years, there has been a notable shift. Women’s health, including the management of menstrual pain (dysmenorrhea), has become a topic of increasing interest and discussion. This shift has been fueled by growing recognition of the importance of addressing women’s unique health needs and the potential for physiotherapy to offer effective, non-invasive treatment options. The expanding body of research has highlighted the role of physiotherapy in this domain, with more studies focusing on the impact of targeted interventions, such as therapeutic exercise, on conditions like dysmenorrhea.

Thus, to highlight the role of physiotherapy, and particularly the role of therapeutic physical exercise, this scoping review aims to synthesize existing evidence regarding therapeutic exercise programs and protocols for managing dysmenorrhea. Specifically, we address two primary research questions:

1. What therapeutic physical exercise programs were used to reduce pain in women with dysmenorrhea?
2. What exercise protocols were used to reduce pain in women with dysmenorrhea?

## 2. Materials and Methods

This scoping review was conducted according to the Joanna Briggs Institute methodology for scoping reviews. Registration was completed on Open Science Framework (OSF) [20]. It followed five steps: identify the research question, identify relevant studies, select the study, map the data, and collate, summarize and report the results. This scoping review was also conducted according to the structure of Preferred Reporting Items for Systematic Reviews and Meta-Analysis extension for Scoping Reviews (PRISMA-ScR) [21].

### 2.1. Eligibility Criteria

The eligibility criteria were established by the acronym PCC (Population, Concept, and Context) according to the methodology of Joanna Briggs Institute (JBI) Reviewer’s Manual for conducting scoping reviews [20]:

- Population: women with dysmenorrhea
- Concept: therapeutic physical exercise program and exercise protocols considered to manage dysmenorrhea
- Context: open context-practice of physical exercise and exercise protocols in outpatient physiotherapy clinics and cabinets, in rehabilitation centers, and home-based settings

### 2.2. Evidence Sources

The search was limited to publications between 1 January 2013 to 30 April 2023, in order to guarantee up-to-date data. As the aim of this study was to collect all the information found on the subject, primary and secondary publications were considered, and qualitative studies, books, book chapters, systematic reviews, meta-analyses and review articles (narrative reviews) were excluded.

### 2.3. Research Strategy

To obtain the most comprehensive search of the available literature, four databases were used: PubMed, ScienceDirect, and Cochrane Library. The collection was carried out by two independent reviewers (PR and SB). To assure the quality of the research, limits were imposed, represented by the terms “AND” and “OR”. The words selected as part of the research expression were: dysmenorrhea, menstrual pain, uterine pain, physical exercise, physical activity, physical exercise program, analgesia and pain relief. After selecting the words according to the PCC strategy, the expression used was as follows: (dysmenorrhea OR period pain OR uterine pain) AND (physical exercises OR physical activities OR physical exercise program) AND (analgesia OR pain relief). Depending on each database, adaptations were made to the research expression, as can be seen in Table 1.

### 2.4. Evidence Selection

**Figure 1:**
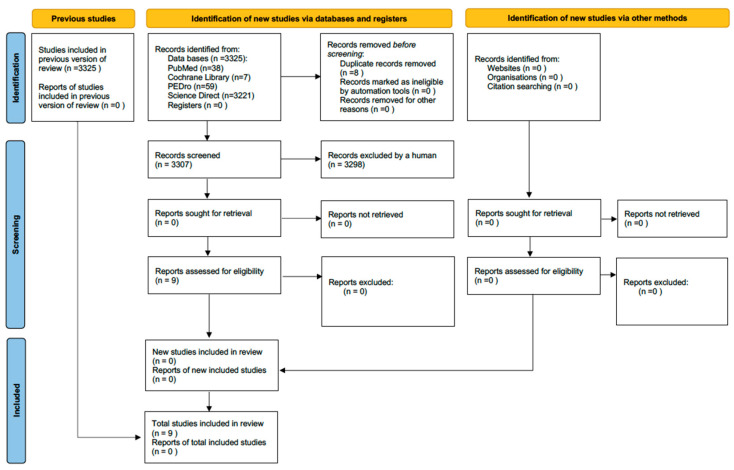
Flowchart of the scoping review according to the PRISMA-ScR model.

The articles were selected in several stages. Articles were excluded based on predefined criteria: unavailable full texts, publication date before January 2023 and did not correspond to the types of studies chosen. Following preliminary screening, all potentially eligible articles were imported into Zotero bibliography management software version 5.0 using Zotero Connector to identify and remove duplicate records across databases. Subsequently, two different reviewers (PR and SB) independently checked the titles of the remaining articles and then the abstracts. Once these steps had been completed, the same two reviewers read all the articles in full to decide whether they could be included in this scoping review. Any disagreement was discussed with the aim of reaching a consensus. If the latter was not reached, a third examiner (SL) was consulted to make the final selection. This selection process took account of the PCC and is detailed in the flowchart PRISMA-ScR (Figure 1) [21].

### 2.5. Data Extraction

After reading the articles, the researchers extracted the relevant data for this scoping review. Firstly, article name, date, and type of study were identified. Subsequently, the objectives of the selected studies were defined to know the intention of the study. So, 2 researchers (PR and SB) extracted information about the study types, study objectives, number of participants, participant age, intervention programs and outcomes.

### 2.6. Analysis and Results Presentation

Results are summarized in tabular format, presenting systematically extracted data categories that address the study objectives and research questions. The data synthesis incorporates key characteristics from included studies to facilitate comprehensive analysis.

## 3. Results

### 3.1. Selection of Evidence Sources

Initially, a total of 3325 studies were identified in the databases: 38 from PubMed, 7 from Cochrane Library, 59 from PEDro and 3221 from ScienceDirect. Duplicate articles were excluded (n = 18), leaving 3307 studies for screening. Two researchers first read the titles of the retained articles followed by the abstracts of the articles selected after reading the titles. After this stage, 3298 articles were excluded since they did not address the relationship between dysmenorrhea and physical exercise, which are the two topics covered by this scoping review, leaving 9 articles for full-text review. No article was excluded after this stage.

### 3.2. Types of Study

After the selection process, 9 articles were included: 4 RCT studies [22,23,24,25], 1 clinical trial study [26], 1 guideline review [27], 1 cross-sectional study [28], 1 experimental study [29] and 1 quasi-experimental study [30].

### 3.3. Objectives

Regarding the objectives of the 9 included studies, it was found that the main objective of all of them was to determine the impact of physical exercise on dysmenorrhea symptoms, whether primary or secondary. Among the 9 included studies, 8 focused on primary dysmenorrhea [22,23,24,25,26,27,29,30] while one examined secondary dysmenorrhea [28]. The exercise interventions varies across studies, with aerobic exercise predominantly used [24,26,29,30]. One study focuses on isometric exercise [22], another in low-intensity exercises (yoga) [25] and the last in a structured FITT (Frequency, Intensity, Time, Type) protocol [23]. On the other hand, the cross-sectional study [28] not only looked at physical exercise, but also at different forms of self-management strategies for endometriosis-related pain. The guideline review [27] aims to investigate and intervene in primary dysmenorrhea. This intervention includes the practice of physical exercise, but this is not the only one. Within the methods studied, exercise is emphasized, but not exclusively.

### 3.4. Participants

The combined sample size across the 9 articles selected for this scoping review comprised 875 women with dysmenorrhea. The guideline review, due to the type of study has no participants. The cross-sectional study [28] has the largest sample, with 484 responses to the questionnaire on physical and/or psychological techniques that women could perform/modify on their own, or lifestyle interventions to reduce symptoms associated with dysmenorrhea. Two studies [25,30] included 30 participants each, and the experimental study [29] included 37 participants. In addition, the other three studies have a considerable sample size, with 68 participants in each [22] and 70 participants in each of the other two [24,26]. Finally, one of the RCTs [23] has 86 participants. The last seven studies described divide their sample into two equal groups, forming an experimental group and a control group. On the other hand, all the studies included in this scoping review exclusively involve women, more specifically women with primary and/or secondary dysmenorrhea. In 8 out of 9 studies, including the guideline review, only women with primary dysmenorrhea were studied [22,23,24,25,26,27,29,30], while only one article included women with secondary dysmenorrhea [28]. Although 5 studies included women aged between 18 and 25 years [22,23,25,29,30], 2 articles included women aged 18 to 45 years [28] and 1 article included women from 41.4 ± 22.25 to 71.4 ± 6.24 years [26].

### 3.5. Intervention Program

The articles selected cover different interventions in different contexts. Four of these studies included aerobic training as an intervention, in a physiotherapy school supervised by a specialized physiotherapist [24], or independently after having received instructions [26], or under the supervision of an experienced physiotherapist [29], or even at home [30]. On the other hand, 3 other articles focused on non-specific exercises, such as isometric exercises [22], or endurance exercises and/or walking [23,28]. The guideline review [27] addresses the issue of exercise in general as a complementary or alternative intervention in reducing menstrual pain. In addition, this guideline review also discusses means of self-care, such as exercise, transcutaneous electrical nerve stimulation (TENS), acupressure, behavioral intervention, topical heat application, dietary supplements, as well as describing medical treatment options, such as drug therapy (non-hormonal therapy/hormonal therapy) and surgical treatment (mainly for secondary dysmenorrhea). Table 2 also shows that two articles address different types of stretching as an intervention, one of them [24] specifically dealing with stretching in the lumbar and pelvic regions. The other article [28] addresses stretching, but also self-care as an intervention. In fact, this article describes different forms of self-care, such as the use of cannabis, acupressure, application of cold or heat, rest, intervention with massage, medicinal herbs and/or changes in diet. It was also noted that two studies used low-intensity exercise as an intervention. One of these two articles [28] approaches the practice of meditation exercises, yoga, Pilates and/or breathing techniques to reduce the pain associated with menstruation. The other article [25] also focuses on yoga and low-intensity exercises, but with a gym ball. Finally, as shown in Table 2, two articles present muscle strengthening as an intervention. One of these [24] mainly focuses on strengthening the abdominal and gluteal muscles, while the other [30] focuses mainly on strengthening the core muscles. In general, these 9 articles address the practice of physical exercise, whatever the modality, to investigate its role in reducing menstrual pain.

### 3.6. Outcomes

Of the nine articles selected, one is a guideline review [27], which therefore does not have a specific outcome. Of the remaining eight, six have the explicit outcome of reducing menstrual pain/dysmenorrhea-related pain, be it primary [22,23,24,25,30] or secondary [28]. The other two articles [26,29] have the common outcome of reducing the specific symptoms of primary dysmenorrhea, which also include menstrual pain. In addition, two articles [22,29] have the additional outcome of improving the emotional/psychological well-being of women with dysmenorrhea, including a wider impact on their overall quality of life. Similarly, addressing the improvement of primary dysmenorrhea more generally, a third article [26] shares this outcome. Finally, there was a third outcome, focused on improving the quality of life of women with dysmenorrhea in two articles [24,29].

## 4. Discussion

The aim of this scoping review was to identify therapeutic physical exercise programs and exercise protocols that have been applied to women with dysmenorrhea, to identify the existing evidence on the subject and determine the studies that have been carried out in this area that needs to be further explored or clarified. The results of this review revealed the scarcity of information available on the subject.

In these 9 selected studies, 8 articles [22,23,24,25,26,27,29,30] evaluated the impact of physical exercise on primary dysmenorrhea, while only 1 article [28] talks about secondary diseases, particularly endometriosis. This scoping review has therefore made it possible to identify the lack of evidence in cases of secondary dysmenorrhea. Future studies should address this condition, with the aim of reducing pain and therefore improving the quality of life of these women. In fact, in many cases, the severity of menstrual pain often results in substantial functional impairment, affecting both occupational and social engagement, with a negative impact on their quality of life [6].

It is also important to know that, on average, a woman waits between 7 to 10 years before being diagnosed with endometriosis [31,32]. It means that for all these years, they faced the symptoms of secondary dysmenorrhea without understanding the real cause, and without knowing how to relieve them. Endometriosis is just one example of a recently recognized condition, and medicine and physiotherapy are paying more attention to it. It is hoped that solutions for pain relief can be found in the future, possibly including therapeutic exercise programs. In fact, increasing research on this subject enhances the likelihood of identifying effective, evidence-based interventions and solutions.

On the other hand, more than half of the 9 articles only involved students in their 20s [22,23,25,29,30]. There is also a lack of evidence regarding the benefits of physical exercise in women over 25 with dysmenorrhea. In the future, it would be beneficial to conduct studies involving menstruating participants from all age groups, to determine whether there are differences in symptoms and interventions according to the stages of female life and the gynecological history of everyone, such as previous pregnancies and the impact of hormonal treatments carried out.

A very important consideration to highlight in this scoping review is the presence of an inconsistency in the age ranges mentioned in one of the included studies [26]. This article includes women ranging from 41.4 ± 22.25 to 71.4 ± 6.24 years, which lacks coherence. It does not seem logical to include women who are already beyond the age of menopause, which usually occurs between 45 and 55 years old [33], in a study on dysmenorrhea and this fact could have potentially skewed the results.

Another variation found is that the studies included in this scoping review studied different types of physical exercise, mainly aerobic exercise [24,26,29,30], but also exercise based on a protocol, the FITT protocol in this case [23], isometric exercise [22], yoga [25], stretching, Tai-chi, Pilates, breathing exercises and meditation [28], acupressure and TENS [27].

These different types of exercises all play a role in reducing pain, offering different benefits. For example, aerobic exercise, such as walking, swimming or cycling, stimulates blood circulation, which results in the release of endorphins and thus a feeling of well-being [14]. Aerobic exercise stimulates the production and release of endorphins—endogenous opioid peptides that act as natural painkillers. Endorphins bind to opioid receptors in the brain and spinal cord, reducing the perception of pain and creating a sense of well-being. This effect is particularly relevant in dysmenorrhea, where endorphin levels are often dysregulated, contributing to heightened pain sensitivity. By increasing endorphin production, aerobic exercise helps mitigate the severity of menstrual pain while also improving mood and reducing stress, which are important factors in pain perception [14,15,16]. These activities not only support physical fitness but also contribute to improved emotional health, which is often affected in individuals experiencing menstrual pain. In turn, stretching exercises can help to relax the abdominal muscles, thus relieving tension and musculoskeletal pain associated with menstrual pain [34]. Strengthening core and lower abdominal muscles can enhance stability and support for the pelvic region, which may reduce muscle tension and cramping associated with dysmenorrhea. Improved muscular endurance can also help mitigate fatigue during menstruation, contributing to better functionality and comfort [34]. In addition, yoga exercises can provide relaxation and emotional management through specific postures, breathing exercises and relaxation techniques [35,36].

Besides the different types of exercise, they also differ in intensity (number of sets, number of repetitions), frequency (number of sessions per week), program duration (number of weeks or months), exercise duration and the period chosen for the exercise (pre-menstrual period, during menstruation or post-menstruation).

Due to the diversity of existing approaches, it has not been possible to reach a definitive conclusion so far, as there is no viable comparison between them. Future investigations should aim to establish more consistent methodologies and explore comparative effectiveness to provide clinicians with evidence-based guidelines for integrating exercise into routine care for individuals with dysmenorrhea. By addressing these gaps, we can enhance our understanding of how exercise can be effectively utilized in clinical practice, ensuring a more targeted and impactful approach to pain management. In clinical practice, physiotherapists can use the existing evidence to tailor exercise programs to individual needs, preferences, and clinical presentations. By continuously evaluating and adapting interventions, clinicians can maximize the therapeutic potential of exercise in pain management while contributing valuable insights to the evolving body of research.

The outcomes of the included studies focus on finding solutions to improve the quality of life and well-being of women with dysmenorrhea. Reduction of menstrual pain and specific dysmenorrhea symptoms was the main outcome in most studies [22,23,24,25,28,30], highlighting the importance of finding effective interventions to relieve pain and improve the quality of life of these women. In addition, two articles [22,29] also explored the impact of interventions on women’s emotional and psychological well-being, highlighting the importance of considering dysmenorrhea-related psychosocial issues. In fact, the psychosocial aspect is often neglected, even though it plays an essential role in pain perception [7]. These results demonstrate a determination to provide care for women with dysmenorrhea, recognizing the importance of an interdisciplinary approach. Collaborating with different health professionals, such as gynecologists, psychologists and physiotherapists, can play a fundamental role in this care, not only because of their expertise, but also because of the psychological and emotional support they offer. This integral and personalized approach makes it possible to meet the needs of each woman and promote positive and durable results in the management of dysmenorrhea [37,38].

A notable limitation identified in this scoping review is the scarcity of articles that provide conclusive evidence regarding the effectiveness of physical exercise in managing pain associated with menstruation. This limitation reflects the relatively nascent stage of research in this area, as the role of physiotherapy in women’s health has only recently gained attention as a critical area of study. Historically, women’s health issues, including menstrual pain, have been under-researched and under-prioritized, contributing to the limited availability of robust and conclusive data. Despite this, the increasing recognition of the importance of addressing women’s unique health needs has sparked a growing interest in physiotherapy interventions, particularly therapeutic physical exercise, as a non-invasive and accessible treatment option. This limitation highlights the need for further high-quality research, including randomized controlled trials and longitudinal studies, to better understand and quantify the impact of physical exercise on dysmenorrhea. Additionally, the lack of standardized exercise protocols in the studies reviewed poses another challenge, as variability in intervention designs and outcomes makes it difficult to draw definitive conclusions. Nonetheless, this scoping review serves as a valuable contribution to the growing body of knowledge in this field. By synthesizing the existing evidence, it underscores the importance of continued investigation into this topic and provides a foundation for future research efforts. As women’s health continues to gain prominence within physiotherapy, it is expected that the gaps identified in this review will inspire more targeted and comprehensive studies, ultimately strengthening the evidence base and advancing clinical practice. In the most severe cases of menstrual pain, the medical treatments commonly proposed are hormone-based [4,6,39]. However, recent research has increasingly demonstrated the detrimental impact that hormone use can have on women’s health and the environment [40,41,42]. These findings are contributing to the increase in discussions and approaches related to women’s health in our society today. It can be concluded that it is mainly for these reasons that the issue of women’s health is receiving greater attention and consideration nowadays.

Advances in medicine and science, and the growing general interest in women’s health, have generated great expectations regarding future studies on the subject. Previous research has indicated that therapeutic physical exercise can reduce some symptoms associated with dysmenorrhea, but the underlying mechanisms are still unclear and there is a scarcity of studies addressing this issue. It is therefore suggested that future studies should aim to determine more specifically the types of exercise that may be most effective, as well as establishing the appropriate parameters such as frequency, intensity and exercise duration. The pursuit of promising research in this field could yield valuable recommendations for women with dysmenorrhea, helping them to better manage their symptoms and ultimately improve their quality of life.

## 5. Conclusions

The findings of this study highlight that aerobic training, particularly among women in their 20s, emerged as the most frequently utilized form of therapeutic physical exercise for alleviating menstrual pain in the studies reviewed. This suggests that aerobic exercise may hold significant promise as a non-pharmacological intervention for managing dysmenorrhea. Despite this, it is important to recognize that a variety of exercise protocols have been explored, reflecting a diversity of approaches within the existing body of research. While the reviewed studies provide preliminary evidence supporting the role of physical exercise in reducing pain associated with menstruation, the variability in exercise modalities, durations, and intensities presents a challenge in drawing definitive conclusions. Moreover, the limited number of high-quality studies that systematically evaluate the effectiveness of these protocols underscores the need for further research. Future investigations should aim to establish standardized exercise protocols, identify the most effective types and doses of exercise, and explore their long-term benefits for individuals with dysmenorrhea. Additionally, studies should consider a broader demographic, including women of varying ages, fitness levels, and menstrual health profiles, to ensure the findings are generalizable and inclusive. Despite these limitations, this study reinforces the potential of therapeutic physical exercise as a valuable tool in managing dysmenorrhea-related symptoms. By emphasizing the importance of continued research, it sets the stage for more robust evidence to guide clinical practice, empowering physiotherapists and healthcare professionals to integrate exercise interventions more effectively into the care of women experiencing menstrual pain.

## Figures and Tables

**Table 1:** Search strategy according to database.

Database	Research Expression	Added Filters
PubMed	(dysmenorrhea OR period pain OR uterine pain) AND (physical exercises OR physical activities OR physical exercise program) AND (analgesia OR pain relief)	- Text availability: Free full text - Article type: Clinical trial; RCT - Publication date: custom range “start date = 1 January 2013”; “end date = 30 April 2023”
ScienceDirect	(dysmenorrhea OR period pain OR uterine pain) AND (physical exercises OR physical activities OR physical exercise program) AND (analgesia OR pain relief)	- Years: 2013 to 2023, included - Article type: research article; case reports; data articles; discussion; editorials; practice guidelines; short communications; others - Access type: “Open access & Opened archive”
PEDro	- Problem: pain - Body part: perineum or genito-urinary system - Therapy: fitness training; strength training - When searching: Match all search terms (AND)	- Methods: practice guideline; clinical trial - Published since: 2013
Cochrane Library	(dysmenorrhea OR period pain OR uterine pain) AND (physical exercises OR physical activities OR physical exercise program) AND (analgesia OR pain relief)	- Date: custom range = 1 January 2013 to 30 April 2023

**Table 2:** Characteristics of eligible studies.

Authors /Year	Study	Objectives	Participants Characteristics	Intervention Program	Outcomes	
Akbaş & Erdem, 2019 [29]	Experimental study	Investigating the effectiveness of an aerobic training program on premenstrual symptoms, menstrual symptoms, emotional state and quality of life in women with primary dysmenorrhea	Sample: - N = 37 women: - N_EG_ = 18 women - N_CG_ = 19 women Age: EG: 21.10 ± 1.59 years CG: 21.20 ± 1.47 years	Program: - GE: aerobic training program - GC: no intervention Aerobic training: - For 4 weeks, 3 times a week, each time for 50 min - Pain intensity assessed using VAS - Emotional state assessed with the BAI - Quality of life assessed using the SF-36 form Context: under the supervision of an experienced physiotherapist	Premenstrual and menstrual symptoms, emotional state and quality of life	
Armour, Sinclair, et al., 2019 [28]	Cross-sectional study	To determine the prevalence of use, safety and self-rated efficacy of common forms of self-management in women with endometriosis	Sample: - N = 484 responses Age: 18–45 years	Program: Cannabis—heat—dietary options (such as gluten-free, vegan)—hemp oil/CBD oil—acupressure—cold—massage—rest—exercise—medicinal herbs—stretching—meditation/breathing—yoga/Pilates—Tai-chi/Qigong Questionnaire: Questionnaire: define physical and/or psychological techniques that women could carry out on their own or lifestyle interventions Context: online questionnaire	Pain	
Azima et al., 2015 [22]	RCT	Investigating the effect of isometric exercises on the intensity and duration of pain and anxiety levels in students with primary dysmenorrhea	Sample: - N = 68 students - N_EG_ = 34 students - N_CG_ = 34 students Age: EG: 21.08 ± 1.21 years CG: 20.73 ± 1.08 years	Program: - EG: isometric exercises - CG: no intervention Isometric exercises: - From the 3rd day of the menstrual cycle - For 8 weeks - Pain intensity assessed using VAS - Anxiety assessed using a questionnaire Context: autonomous exercises by students from Shiraz University; support from a conductor specializing in rehabilitation	Pain and anxiety	
Burnett & Lemyre, 2017 [27]	Guideline review	Investigations and treatment of primary dysmenorrhea	N/A	Program: Medical treatment: non-hormonal therapy (acetaminophen; non-steroidal anti-inflammatory drugs)—hormonal treatment (combined hormonal contraceptives; progesterone regimens) Complementary and alternative therapy: exercise—TENS—acupuncture/acupressure—behavioral intervention—topical heat—food choices Surgical management: laparoscopy—treatment for endometriosis—conservative surgical procedures—surgical options in the absence of visual abnormalities Context: N/A	N/A	
Dehnavi et al., 2018 [26]	Clinical trials	Investigating the effect of regular aerobic exercise on the severity of primary dysmenorrhea	Sample: - N = 70 women - N_EG_ = 35 women - N_CG_ = 35 women Age: EG: 41.4 ± 22.25 years CG: 71.4 ± 6.24 years	Program: - EG: aerobic exercise - CG: no intervention Aerobic exercise: - For 8 weeks, 3 times a week, for 30 min - Visual pain questionnaire completed by the 2 groups in the first 3 days of the menstrual cycle Context: wherever the participant wants to train, from CD and educational posters containing all the movements performed, handed out at the end of the learning session	Menstrual symptoms: mainly pain, but also nausea and vomiting, bruising and headache, and a general malaise	
Heidarimoghadam et al., 2019 [23]	RCT	To investigate the effects of exercise based on a specific protocol on the severity and duration of primary dysmenorrhea	Sample: - N = 86 students - N_EG_ = 43 students - N_CG_ = 43 students Age: 18–24 years	Program: - EG: FITT protocol-based exercise - CG: physical education class Exercise based on FITT protocol: - For 8 weeks, each with 3 sessions (24 sessions) - Sports protocol proposed by the ACSM with four pillars: frequency of sports sessions, exercise intensity, exercise time and type of exercise. Physical education class: - Once a week, they also do group exercises (volleyball, badminton) for 1 h 30 Context: in gyms using a program that was taught by a sports instructor and a trained researcher	Pain	
Kannan et al., 2019 [24]	RCT	To evaluate the effectiveness of an aerobic treadmill exercise intervention on the pain and symptoms associated with primary dysmenorrhea	Sample: - N = 70 women - N_GE_ = 35 women - N_GC_ = 35 women Age: 18–43 years	Program: - EG: regular aerobic exercise - CG: usual care Aerobic training: - On the treadmill - For 4 weeks, 3 times a week - 70 to 85% of maximum heart rate - Preceded by 10-min warm-up exercises - Followed by relaxation exercises for 10 min, including stretches for the lower back, stretches for the pelvic region and strengthening of the abdominal and gluteal muscles - Exercise between periods, no exercise during the week of menstruation Context: at the Otago University School of Physiotherapy with a registered physiotherapist with 3 years’ experience specializing in pain and women’s health, and at home	Pain and quality of life	
Sandhiya M et al., 2020 [30]	Quasi-experimental study	To evaluate and compare the effect of aerobic exercise and core strengthening in primary dysmenorrhea	Sample: - N = 30 students - N_GA_ = 15 students - N_GB_ = 15 students Age: 18–25 years	Program: - GA: aerobic exercise - GB: core strengthening exercise with a small stability ball Aerobic exercise: - For 8 weeks, 3 times a week, each time for 40 min - Measuring instruments: VMSS and MMDQ Context: at home	Pain and quality of life	
Padmanabhan, K, et al., 2018 [25]	RCT	Comparing the effectiveness of yoga asana and gym ball/therapy ball/swiss ball exercises in treating women with primary dysmenorrhea	Sample: - N = 30 women - N_GA_ = 15 women - N_GB_ = 15 women Age: GA: 20.2 ± 3.8 years GB: 20.7 ± 3.1 years	Program: - GA: yoga asana classes - GB: exercises with a gym ball/therapy ball/swiss ball Context: supervised during the first session by qualified people with more than 5 years’ experience in yoga; unsupervised, in autonomy (at home), during the other sessions	Pain associated with primary dysmenorrhea	

BAI = Beck Anxiety Inventory; CBD = cannabidiol; VAS = Visual Analogue Scale; CG = control group; EG = experimental group; N = total; SF-36 = Short-Form 36; N/A = not applicable; TENS = Transcutaneous Electrical Nerve Stimulator; ACSM = American College of Sports Medicine; CD = Compact Disc; FITT = Frequency, Intensity, Time, Type; MMDQ = Moos Menstrual Distress Questionnaire; VMSS = Verbal Multidimensional Scoring System.

## Data Availability

The data presented in this study are available on request from the corresponding author.

## References

[1] Bernardi M., Lazzeri L., Perelli F., Reis F.M., Petraglia F. (2017). Dysmenorrhea and related disorders. F1000Res.

[2] Wong C.L., Farquhar C., Roberts H., Proctor M. (2009). Oral contraceptive pill for primary dysmenorrhoea. Cochrane Database Syst. Rev..

[3] Armour M., Smith C.A., Steel K.A., Macmillan F. (2019). The effectiveness of self-care and lifestyle interventions in primary dysmenorrhea: A systematic review and meta-analysis. BMC Complement. Altern. Med..

[4] Guimarães I., Póvoa A.M. (2020). Primary Dysmenorrhea: Assessment and Treatment. Rev. Bras. Ginecol. Obstet..

[5] Koninckx P.R., Fernandes R., Ussia A., Schindler L., Wattiez A., Al-Suwaidi S., Amro B., Al-Maamari B., Hakim Z., Tahlak M. (2021). Pathogenesis Based Diagnosis and Treatment of Endometriosis. Front. Endocrinol..

[6] Osayande A.S., Mehulic S. (2014). Diagnosis and initial management of dysmenorrhea. Am. Fam. Physician.

[7] Baker F.C., Driver H.S., Rogers G.G., Paiker J., Mitchell D. (1999). High nocturnal body temperatures and disturbed sleep in women with primary dysmenorrhea. Am. J. Physiol..

[8] Iacovides S., Avidon I., Baker F.C. (2015). What we know about primary dysmenorrhea today: A critical review. Hum. Reprod. Update.

[9] McKenna K.A., Fogleman C.D. (2021). Dysmenorrhea. Am. Fam. Physician.

[10] MacKichan F., Paterson C., Henley W.E., Britten N. (2011). Self-care in people with long term health problems: A community based survey. BMC Fam. Pract..

[11] Armour M., Dahlen H.G., Smith C.A. (2016). More Than Needles: The Importance of Explanations and Self-Care Advice in Treating Primary Dysmenorrhea with Acupuncture. Evid. Based Complement. Alternat Med..

[12] French L. (2005). Dysmenorrhea. Am. Fam. Physician.

[13] Ziaei S., Zakeri M., Kazemnejad A. (2005). A randomised controlled trial of vitamin E in the treatment of primary dysmenorrhoea. BJOG.

[14] Amorosi M. (2014). Correlation between sport and depression. Psychiatr. Danub..

[15] Anderson E., Shivakumar G. (2013). Effects of exercise and physical activity on anxiety. Front. Psychiatry.

[16] Harber V.J., Sutton J.R. (1984). Endorphins and exercise. Sports Med..

[17] Bielecki J.E., Tadi P. (2023). Therapeutic Exercise.

[18] Caspersen C.J., Powell K.E., Christenson G.M. (1985). Physical activity, exercise, and physical fitness: Definitions and distinctions for health-related research. Public. Health Rep..

[19] Berghmans B. (2018). Physiotherapy for pelvic pain and female sexual dysfunction: An untapped resource. Int. Urogynecol J..

[20] Peters M.D.J., Marnie C., Tricco A.C., Pollock D., Munn Z., Alexander L., McInerney P., Godfrey C.M., Khalil H. (2020). Updated methodological guidance for the conduct of scoping reviews. JBI Evid. Synth..

[21] Tricco A.C., Lillie E., Zarin W., O’Brien K.K., Colquhoun H., Levac D., Moher D., Peters M.D.J., Horsley T., Weeks L. (2018). PRISMA Extension for Scoping Reviews (PRISMA-ScR): Checklist and Explanation. Ann. Intern. Med..

[22] Azima S., Bakhshayesh H.R., Abbasnia K., Kaviani M., Sayadi M. (2015). Effect of Isometric Exercises on PrimaryDysmenorrhea: A Randomized Controlled Clinical Trial. GMJ.

[23] Heidarimoghadam R., Abdolmaleki E., Kazemi F., Masoumi S.Z., Khodakarami B., Mohammadi Y. (2019). The Effect of Exercise Plan Based on FITT Protocol on Primary Dysmenorrhea in Medical Students: A Clinical Trial Study. J. Res. Health Sci..

[24] Kannan P., Chapple C.M., Miller D., Claydon-Mueller L., Baxter G.D. (2019). Effectiveness of a treadmill-based aerobic exercise intervention on pain, daily functioning, and quality of life in women with primary dysmenorrhea: A randomized controlled trial. Contemp. Clin. Trials.

[25] Padmanabhan K., Sudhakar S., Aravind S., Kumar C.P., Monika S. (2018). Efficacy of Yoga Asana and Gym Ball Exercises in the management of primary dysmenorrhea: A single-blind, two group, pretest-posttest, randomized controlled trial. CHRISMED J. Health Res..

[26] Dehnavi Z.M., Jafarnejad F., Kamali Z. (2018). The Effect of aerobic exercise on primary dysmenorrhea: A clinical trial study. J. Educ. Health Promot..

[27] Burnett M., Lemyre M. (2017). No. 345-Primary Dysmenorrhea Consensus Guideline. J. Obstet. Gynaecol. Can..

[28] Armour M., Sinclair J., Chalmers K.J., Smith C.A. (2019). Self-management strategies amongst Australian women with endometriosis: A national online survey. BMC Complement. Altern. Med..

[29] Akbaş E., Erdem E.U. (2019). Effectiveness of Group Aerobic Training on Menstrual Cycle Symptoms in Primary Dysmenorrhea. Med. J. Bakirkoy.

[30] Sandhiya M., Senthil Selvam P., Manoj Abraham M., Palekar T.J., Sundaram M.S., Priya K., Christina J. (2020). A Study To Compare The Effects Of Aerobic Exercise Versus Core Strengthening Exercise Among College Girls With Primary Dysmenorrhea. Int. J. Res. Pharm. Sci..

[31] Hudelist G., Fritzer N., Thomas A., Niehues C., Oppelt P., Haas D., Tammaa A., Salzer H. (2012). Diagnostic delay for endometriosis in Austria and Germany: Causes and possible consequences. Hum. Reprod..

[32] Staal A.H., van der Zanden M., Nap A.W. (2016). Diagnostic Delay of Endometriosis in the Netherlands. Gynecol. Obstet. Investig..

[33] Parazzini F., Di Martino M., Pellegrino P. (2017). Magnesium in the gynecological practice: A literature review. Magnes. Res..

[34] Lorena S.B., Lima M.C., Ranzolin A., Duarte Â.L. (2015). Effects of muscle stretching exercises in the treatment of fibromyalgia: A systematic review. Rev. Bras. Reumatol..

[35] Saeed S.A., Cunningham K., Bloch R.M. (2019). Depression and Anxiety Disorders: Benefits of Exercise, Yoga, and Meditation. Am. Fam. Physician.

[36] Saud A., Abbasi M., Merris H., Parth P., Jones X.M., Aggarwal R., Gupta L. (2022). Harnessing the benefits of yoga for myositis, muscle dystrophies, and other musculoskeletal disorders. Clin. Rheumatol..

[37] Choi B.C., Pak A.W. (2007). Multidisciplinarity, interdisciplinarity, and transdisciplinarity in health research, services, education and policy: 2. Promotors, barriers, and strategies of enhancement. Clin. Investig. Med..

[38] Körner M. (2010). Interprofessional teamwork in medical rehabilitation: A comparison of multidisciplinary and interdisciplinary team approach. Clin. Rehabil..

[39] Vercellini P., Buggio L., Frattaruolo M.P., Borghi A., Dridi D., Somigliana E. (2018). Medical treatment of endometriosis-related pain. Best Pract. Res. Clin. Obstet. Gynaecol..

[40] Cagnacci A. (2017). Hormonal contraception: Venous and arterial disease. Eur. J. Contracept. Reprod. Health Care.

[41] Skovlund C.W., Mørch L.S., Kessing L.V., Lidegaard Ø. (2016). Association of Hormonal Contraception With Depression. JAMA Psychiatry.

[42] Zuercher B. (2022). Impact des médicaments sur l’environnement. Rev. Med. Suisse.

